# Tailoring treatment: dog breed status influences pain assessment and treatment in emergency veterinary care

**DOI:** 10.3389/fpain.2025.1589082

**Published:** 2025-11-17

**Authors:** Rachel M. P. Caddiell, Philip A. White, Eleanor H. McNamee, Alex M. Lynch, B. Duncan X. Lascelles, Margaret E. Gruen

**Affiliations:** 1 Comparative Behavioral Research and Thinking Pets Program, Department of Clinical Sciences, College of Veterinary Medicine, North Carolina State University, Raleigh, NC, United States; 2 Translational Research in Pain, Department of Clinical Sciences, College of Veterinary Medicine, North Carolina State University, Raleigh, NC, United States; 3 Berry Consultants, Austin, TX, United States; 4 Department of Statistics, College of Physical and Mathematical Sciences, Brigham Young University, Provo, UT, United States; 5 Emergency Critical Care, Department of Clinical Sciences, College of Veterinary Medicine, North Carolina State University, Raleigh, NC, United States; 6 Comparative Pain Research and Education Centre, Department of Clinical Sciences, College of Veterinary Medicine, North Carolina State University, Raleigh, NC, United States; 7 Center for Translational Pain Research, Department of Anesthesiology, Duke University, Durham, NC, United States; 8 Thurston Arthritis Center, UNC, Chapel Hill, NC, United States

**Keywords:** canine pain assessment, dog pain management, veterinary emergency care, breed differences, retrospective study

## Abstract

**Background:**

Several studies have demonstrated that veterinarians hold breed-specific beliefs about canine pain sensitivity. However, it remains unknown whether these beliefs impact how veterinarians recognize and treat pain in a clinical setting. Therefore, the objective of this study was to determine if there were differences in the assessment and treatment of pain across patients admitted to a veterinary emergency room (ER) from different breeds.

**Methods:**

Veterinary ER records were retrospectively analyzed to evaluate the effects of breed on the assessment and treatment of pain in canine patients admitted to a single academic ER over a two-year period. Extracted data included patient signalment and information documented in medical evaluations completed by ER clinicians.

**Results:**

The final sample included records from 3,744 patients across 69 breeds/breed types. Patient breed and the service the patient was transferred to from the ER were significantly explanatory for differences observed in pain scores and pain management plans assigned. The effect of breed and transfer service remained robust when accounting for covariates.

**Conclusions and clinical relevance:**

Certain breeds were assigned pain scores lower than average, while other breeds were assigned higher than average pain scores despite a lack of evidence that these breeds presented with less or more painful conditions. As breed-specific beliefs do not align with experimental measures of pain sensitivity, the present findings have implications to help refine pain education and medical decision-making and ultimately improve patient care.

## Introduction

Within human medicine, disparities related to the recognition, treatment and management of patient pain have been widely documented (1–6). These disparities in care have been attributed to systemic issues, such as access to healthcare (7, 8), as well as other non-medical factors including health care workers’ beliefs about pain sensitivity in others (5, 8–10). Numerous studies have demonstrated that healthcare workers report different pain ratings for patients perceived as belonging to different races, ethnicities, genders, socioeconomic statuses, and ages—including pediatric and geriatric patients (11–18). Additionally, prior research has identified that minority patients are less likely to be provided with pain management (19–24) and less likely to be treated with opioids for pain (20, 21, 24–28). When minority patients are prescribed pain management, they often receive lower doses of analgesics (22, 23, 25, 29–31).

Beliefs about pain sensitivity could also impact treatment in other species, even within one species. Recent research by Gruen and colleagues (32) revealed that veterinarians endorse breed-specific beliefs about pain sensitivity in dogs. Their study surveyed over 1,000 veterinarians and asked them to rate pain sensitivity for 28 different dog breeds. Veterinarians reported distinct pain sensitivity ratings for each breed and these ratings were highly consistent among respondents, indicating a great deal of agreement among the profession. This finding was remarkable because at the time of the survey, there was no existing scientific evidence that would suggest dog breeds differ in pain sensitivity. Follow up studies have demonstrated that veterinary education and clinical experiences contribute to the development of these breed-specific beliefs (33, 34). However, it remains unknown if breed-specific beliefs about canine pain influence veterinarians’ estimations of patient pain and pain management treatment recommendations within a clinical setting.

Therefore, using a retrospective analysis of veterinary hospital records, the objective of this study was to determine if there are differences in the assessment and treatment of pain across patients of different breeds. If differences were found by breed, other factors were explored that may explain the relationships identified. We hypothesized that patients belonging to breeds that veterinarians previously rated as having low pain sensitivity (32, 34) would have lower pain scores and be less likely to be assigned a pain management plan.

## Materials and methods

Records of dogs admitted to the NC State University Veterinary Hospital through the Emergency Room (ER) between January 2017 and December 2018 were accessed. As per NCSU procedure, each patient hospitalized by the ER has a transfer sheet that summarizes their clinical data for the hospital service assuming care of the case (Supplementary Figure S1). Using these transfer documents, data extracted were the patient’s signalment (breed/breed type, purebred or mixed breed status, age, sex, neuter status) and information from the medical evaluation completed by the ER clinician (weight, body condition score, presenting condition, pain score, whether they received a pain management plan and if so, whether their pain management plan included the use of an opioid and/or non-steroidal anti-inflammatory drug (NSAID), and what service they were transferred to). The gender of the ER clinician who treated the patient was noted.

### Primary outcome measures

The primary outcome measures included the pain score assigned to the patient and whether a pain management plan was provided (yes/no) by the ER clinician who examined the patient. Emergency room clinicians assigned pain scores on a 5-point scale ranging from 0 to 4 with higher scores indicating a greater intensity of pain (Supplementary Figure S2). Details of the training protocol are further provided in the Supplementary Materials. Additional outcome measures included whether an opioid (yes/no) or NSAID (yes/no) were prescribed.

### Independent variables of interest: breed and transfer service

The patient's breed/breed type and transfer service (where the patient was transferred to from the ER) were the independent variables of interest. Breed labels used at patient intake that had the same meaning, and/or breeds that shared phenotypic traits and similar health risks (such as the American Springer Spaniel and English Springer Spaniel) were combined in the final data set to condense breed labels (Supplementary Table S1). As Pitbull represents a breed type rather than a singular breed, the following breed labels documented in patient medical records were combined: Pitbull, American Bulldog, American Staffordshire Terrier, Staffordshire Bull Terrier. This was important to include as this breed type is frequently referenced regarding their pain sensitivity (32, 34–37). Transfer service was included as an independent variable to account for presenting condition, based on the assumption that the receiving service corresponded to the dog's initial condition and associated pain level at the time of ER presentation.

### Key covariates

Covariates evaluated included patient sex (male, female), age group (juvenile, adult, senior), body condition score (underconditioned, ideal, over conditioned), neuter status (yes/no), weight (kg), mixed breed status (yes/no), and the ER clinician's gender (male, female).

As absolute age has different clinical importance for dogs of different breeds and sizes, each patient's fractional lifespan age was also calculated by dividing their breed life expectancy from their recorded age. Breed life expectancy was calculated using average breed height and average breed weight (38). Using their fractional lifespan age, each patient was classified into one of three age groups: juvenile (≤0.25), adult (0.26–0.74) or senior (≥0.75). Patient body condition score was assigned by ER clinicians using a 9-point scale.

As clinician gender has been previously demonstrated to impact pain scoring in canine patients (39–42), clinician gender was included as a covariate.

Additionally, the patient's pain score was included as a covariate when evaluating whether a pain management plan was provided, and whether an opioid (yes/no) or NSAID (yes/no) was prescribed.

### Inclusion criteria

If a dog visited the ER multiple times within a 3-month period, only their first visit was retained in the final data set. As the primary goal of this study was to determine whether breed status explained differences in pain scoring and pain management provided to patients in the ER, a decision was made to include breeds/breed types that had 20 or more dogs represented. To explore the effect of breed status within a transfer service, breeds/breed types were included for analysis if at least 5 dogs from a breed were transferred to the same service.

### Data completeness

If breed status was not documented on the patient's ER transfer sheet, the patient's medical records were pulled from the hospital record system to confirm their breed status. If a determination could not be made, the patient was removed from the dataset. When information (e.g., pain score, BCS) was missing from the patient's ER transfer sheet, this was denoted as “not reported”.

### Secondary evaluation: condition-based pain assessment

To answer the question of whether any differences found may be simply due to the fact that certain breeds/breed types present more frequently to the ER with painful conditions, three independent clinicians scored a subset of cases (*N* = 744 across 5 breeds). The five breeds chosen to evaluate in this subset were Cavalier King Charles Spaniel, Dachshund, Chihuahua, German Shepherd and Golden Retriever as these breeds represent a range of pain scores and pain management plans assigned by ER clinicians (see Results section for further details). The clinicians were only provided with the presenting complaint information for these cases and were instructed to determine whether these conditions were painful or not (yes/no).

### Statistical analysis

Statistical analyses for the primary outcome measures were performed using R software (R Core Team). Cumulative link (logit) regression models were used for ordinal variables and logistic regression models were used for binary variables.

Cumulative logit models were used to evaluate the effect of breed and transfer service on pain scores. Additional cumulative logit models were used to evaluate the effect of breed and transfer service on pain scores accounting for the following covariates: patient sex, age group, body condition score, neuter status, weight, mixed breed status and ER clinician's gender.

Logistic regression models were fitted to determine whether breed and transfer service influenced whether a patient was assigned a pain management plan or prescribed opioids. In addition to the previously mentioned covariates, pain score was included as a covariate in these models. To understand whether breed effects remained for differences in pain management plans within patients transferred to the same hospital service, logistic regression models were used to evaluate whether there was an effect of breed within individual services. Due to the sample size and inclusion criteria, breed within transfer service was only examined for internal medicine, neurology, soft tissue surgery, and triage. Only 104 patients were prescribed NSAIDs, therefore, no further analyses were performed for this variable.

A Chi-square test of independence was conducted to examine the association between five breeds and their total yes/no responses for whether the patient's presenting condition was painful. This was performed for each clinician rater. Additional Chi-square tests of independence were performed to determine if there was agreement among clinician raters for the presenting conditions that each breed visited the ER. These statistical analyses were performed using JMP® Pro 16 (SAS Institute Inc., Cary, NC, USA).

*P*-values are reported as summary statistics and should be interpreted with caution to avoid making binary decisions about statistical significance.

## Results

Data were initially extracted from 4,439 ER transfer sheets. After applying inclusion criteria, the final sample included 3,744 patients across 69 breeds/breed types (Table 1). In the final data set, patient cases were transferred from the ER department to one of nine different hospital services (Table 2).

**Table 1:** Dog breeds/breed types included in the final data set.

Dog breed–Number	
Australian Cattle Dog—15^a^	Japanese Chin—5^a^
Australian Shepherd—46	Labradoodle—44
Bassett Hound—24	Labrador Retriever—387
Beagle—120	Lhasa Apso—15
Belgian Malinois—12^a^	Maltese—105
Bernese Mountain Dog—17^a^	Miniature Dachshund—12^a^
Bichon Frise—34	Miniature Pinscher—24
Border Collie—38	Miniature Poodle—71
Boston Terrier—54	Miniature Schnauzer—66
Boxer—123	Mixed Breed Dog—322
Brittany Spaniel—10^a^	Newfoundland—10^a^
Bulldog—44	Papillion—8^a^
Cairn Terrier—12^a^	Pekingese—30
Cavalier King Charles Spaniel—58	Pitbull—139
Chesapeake Bay Retriever—7	Pointer—11^a^
Chihuahua—139	Pomeranian—60
Chow Chow—10^a^	Poodle (Standard)—36
Cocker Spaniel—58	Pug—58
Coonhound—29	Rat Terrier—20
Corgi—33	Rhodesian Ridgeback—13
Dachshund—253	Rottweiler—31
Doberman Pincher—39	Shetland Sheepdog—25
Fox Terrier—11^a^	Shih Tzu—100
French Bulldog—57	Siberian Husky—47
German Shepherd—154	Silky Terrier—6^a^
German Shorthaired Pointer—13^a^	Springer Spaniel—20
Golden Retriever—140	Terrier—39
Goldendoodle—43	Toy Poodle—45
Great Dane—39	Viszla—12^a^
Great Pyrenees—19^a^	Weimaraner—23
Greyhound—21	West Highland White Terrier—24
Havanese—13^a^	Wheaton Terrier—8^a^
Hound—30	Whippet—6^a^
Irish Wolfhound—7	Yorkshire Terrier—139
Jack Russell Terrier—61	

a Indicates a breed that was not included in statistical analyses that required ≥20 dogs per breed.

**Table 2:** Services patients were transferred to from the ER.

Service–Number	
Cardiology—228	Ophthalmology—31
Dentistry—13	Orthopedic surgery—31
Internal medicine—1,492	Soft tissue surgery—423
Neurology—928	Triage—428
Oncology^a^—123	

a Oncology includes patients transferred to both medical oncology and radiation oncology hospital services.

### Primary outcome measures

**Figure 1:**
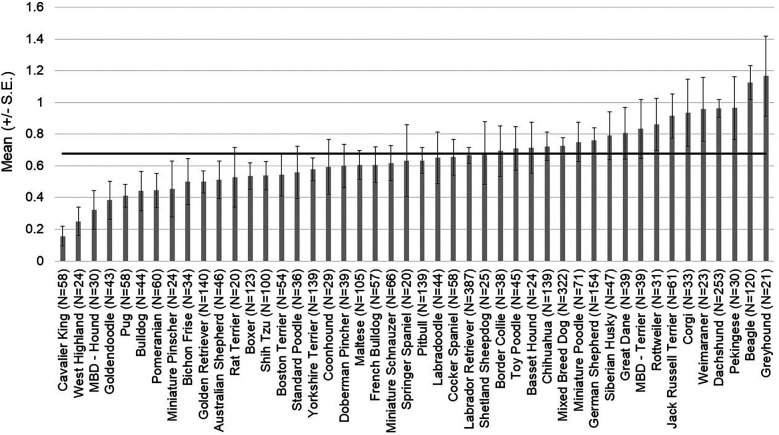
Average pain scores assigned to patients by emergency room clinicians. The mean and SD values are displayed for each breed/breed type. The horizontal line represents the average pain score across breeds for this population of patients.

**Figure 2:**
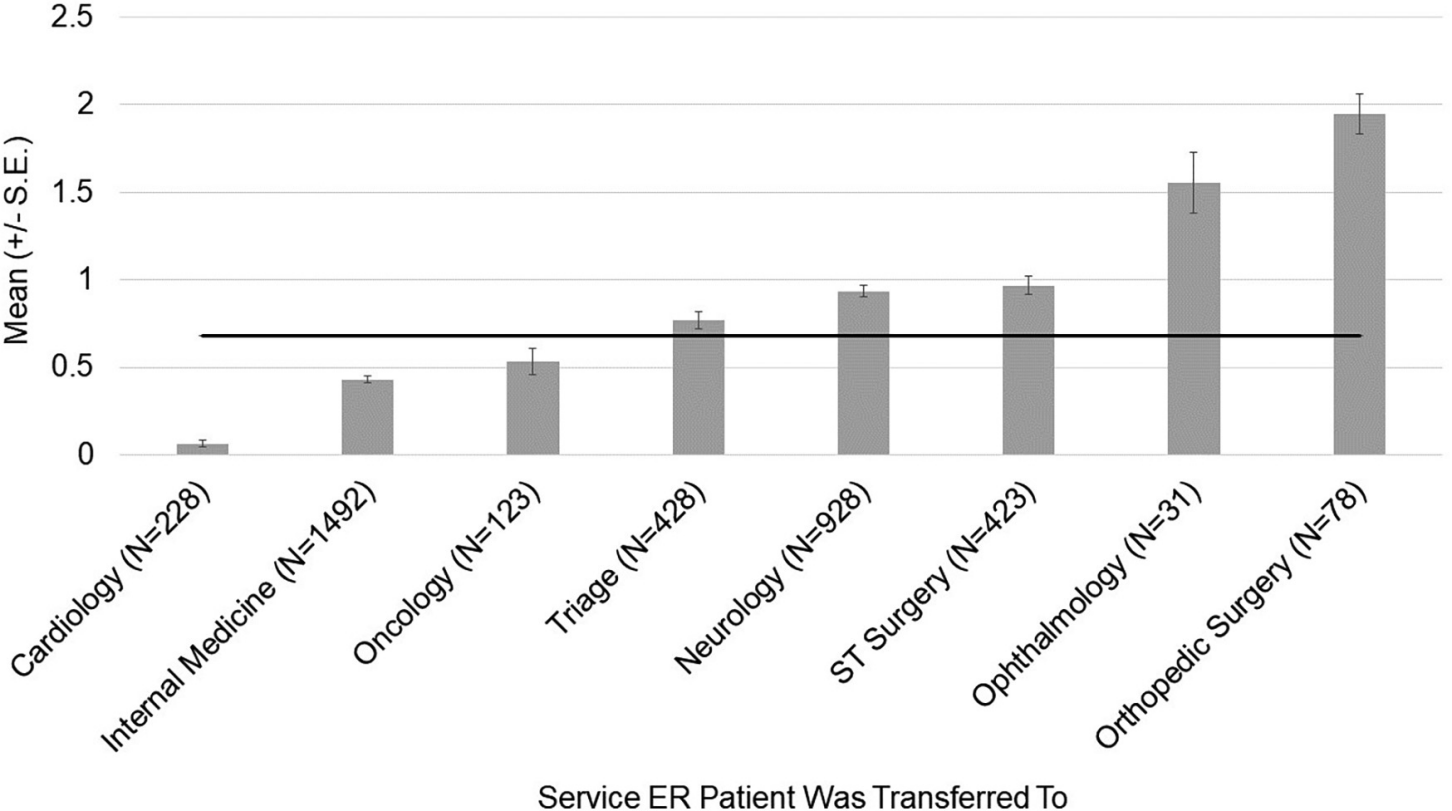
Average pain scores assigned to patients by emergency room clinicians. The mean and SD values are displayed for each hospital service the patient was transferred to. The horizontal line represents the average pain score across hospital services for this population of patients.

For pain scores, there was an effect of breed [*χ*^2^ (45) = 151.14, *p* = 2.136 × 10^−13^] (Figure 1) and transfer service [*χ*^2^ (7) = 329.63, *p* < 2.2 × 10^−16^] (Figure 2). The effect of breed [*χ*^2^ (42) = 147.840, *p* = 1.048 × 10^−13^] and transfer service [*χ*^2^ (7) = 280.224, *p* < 2.2 × 10^−16^] on pain scores remained robust when covariates were considered in the model. Clinician gender emerged as an important covariate [*χ*^2^ (1) = 11.446, *p* = 7.166 × 10^−4^]. Male clinicians rated dogs as having lower pain scores compared to female clinicians (OR = 0.719, z = −3.365, *p* = 7.66 × 10^−4^).

**Figure 3:**
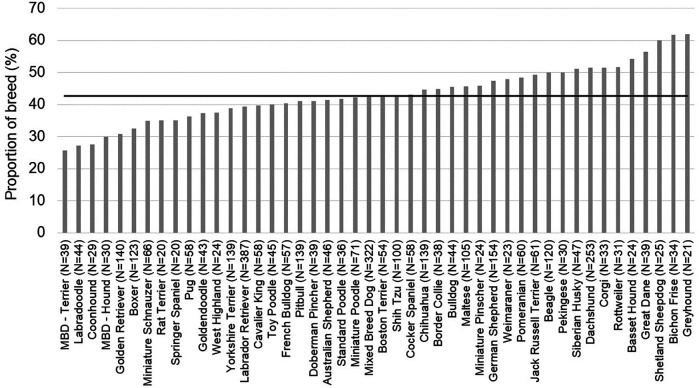
Proportion of patients assigned a pain management plan by emergency room clinicians for each breed represented in the population. The horizontal line represents the average proportion of patients assigned pain management plans for this population of patients.

**Figure 4:**
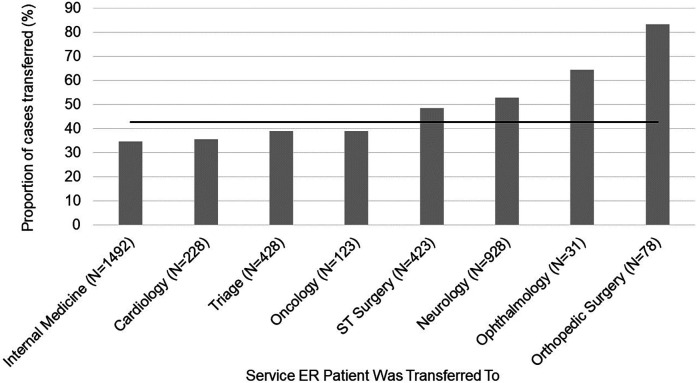
Proportion of patients assigned a pain management plan by emergency room clinicians displayed across hospital services the patient was transferred to from the emergency room. The horizontal line represents the average proportion of patients assigned pain management plans for this population of patients.

Breed [*χ*^2^ (45) = 71.003, *p* = 8.01 × 10^−3^] (Figure 3) and transfer service [*χ*^2^ (7) = 134.623, *p* < 2 × 10^−16^] (Figure 4) were significantly explanatory for whether a pain management plan was assigned to a patient in the ER. The effect of breed [*χ*^2^ (42) = 70.69, *p* = 3.664 × 10^−3^] and transfer service [*χ*^2^ (7) = 118.09, *p* < 2.2 × 10^−16^] on pain management plan remained robust when covariates were considered in the model. Pain score assigned was identified as a crucial covariate [*χ*^2^ (4) = 391.83, *p* < 2.2 × 10^−16^]. Patients who were assigned a higher pain score were more likely to receive a pain management plan (OR_1|0_ = 4.35, *p* < 0.001; OR_2|0_ = 8.12, *p* < 0.001; OR_3|0_ = 11.49, *p* < 0.001; OR_4|0_ = 12.85, *p* < 0.001). Other key covariates that emerged included patient's age group [*χ*^2^ (2) = 13.77, *p* < 1.023 × 10^−3^] and body condition score [*χ*^2^ (1) = 8.25, *p* < 4.081 × 10^−3^]. Senior dogs were more likely than adult dogs to receive a pain management plan (OR = 1.376, z = 3.102, *p* = 1.92 × 10^−3^). Patients with a higher body condition score had a higher likelihood of receiving a pain management plan (OR = 1.095, z = 2.187, *p* = 0.03). Within transfer services, breed had limited explanatory power for most services (*p* > 0.05). However, breed did explain differences in pain management plans assigned for patients transferred to neurology [*χ*^2^ (36) = 72.667, *p* < 2.832 × 10^−4^] (Supplementary Figure S3) and triage [*χ*^2^ (23) = 39.861, *p* = 0.012] (Supplementary Figure S4).

**Figure 5:**
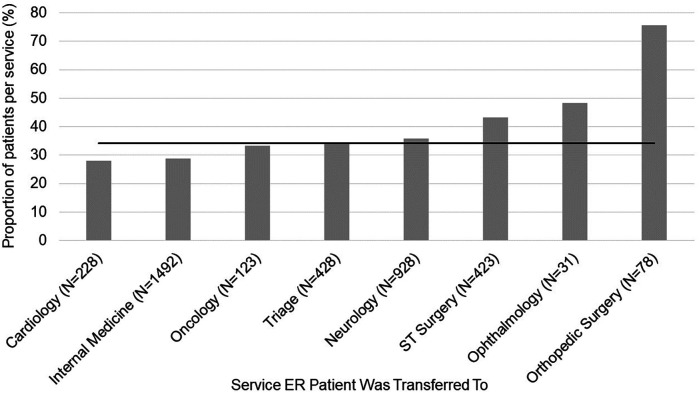
Proportion of patients prescribed an opioid by emergency room clinicians displayed across hospital services the patient was transferred to from the emergency room. The horizontal line represents the average proportion of patients prescribed an opioid for this population of patients.

Breed did not explain whether patients were assigned opioids in the ER (*p* > 0.05). Still, there was an effect of transfer service [*χ*^2^ (7) = 89.758, *p* < 2 × 10^−16^] (Figure 5). The effect of transfer service [*χ*^2^ (7) = 69.43, *p* = 0.027] persisted when covariates were considered in the model. Once again, pain score proved to be a significant covariate [*χ*^2^ (4) = 420.82, *p* < 2.2 × 10^−16^]. Patients who were assigned a higher pain score were more likely to receive an opioid (OR_1|0_ = 3.88_,_ *p* < 0.001; OR_2|0_ = 10.27_,_ *p* < 0.001; OR_3|0_ = 13.26_,_ *p* < 0.001; OR_4|0_ = 21.18, *p* < 0.001). These are interpreted as the odds of receiving an opioid at each pain score (1, 2, 3, or 4) compared to a pain score of zero. Additionally, body condition score was identified as an important covariate [*χ*^2^ (1) = 11.46, *p* = 7.123 × 10^−4^]. Patients with a higher body condition score were more likely to be prescribed opioids (OR = 1.131, z = 2.871, *p* = 0.004).

### Secondary evaluation: condition-based pain assessment

The Pearson Chi-Square was significant for all three raters [Rater 1: *χ*² (4) = 48.403, *p* < 0.001; Rater 2: *χ*² (4) = 80.819, *p* < 0.001; Rater 3: *χ*² (4) = 67.835, *p* < 0.001] suggesting that breeds presented to the ER for different medical conditions with some breeds presenting to the ER more frequently for conditions that each rater deemed as painful. However, raters differed in which conditions they perceived as painful and therefore, differed in their ratings of what conditions were painful for four out of the five breeds [Cavalier King Charles Spaniel: *χ*² (2) = 10.972, *p* = 4.1 × 10^−3^; Dachshund: *χ*² (2) = 10.194, *p* = 6.1 × 10^−3^; German Shepherd: *χ*² (2) = 7.735, *p* = 2.09 × 10^−2^; Golden Retriever: *χ*² (2) = 40.171, *p* < 1 × 10^−4^]. For example, Rater 2 scored 29.3% of Golden Retrievers’ presenting conditions as painful whereas Rater 3 scored 66.4% of Golden Retrievers’ presenting conditions as painful (Table 3).

**Table 3:** Proportion of breed rated as having a painful condition based on their presenting complaint by three independent clinician raters.

Breed	Rater 1	Rater 2	Rater 3
Cavalier King Charles Spaniel	37.9% (22/58)	15.5% (9/49)	15.5% (9/49)
Chihuahua	53.2% (74/139)	41.7% (58/139)	41.0% (57/139)
Dachshund	77.5% (196/253)	66.4% (168/253)	66.0% (167/253)
Golden Retriever	54.3% (76/140)	29.3% (41/140)	66.4% (93/140)
German Shepherd	59.1% (91/154)	44.8% (69/154)	57.8% (89/154)

Raw values are presented in parentheses.

## Discussion

The results showed that that patient breed and the service the patient was transferred to from the ER were significantly explanatory for differences observed in pain scores and pain management plans assigned. Additionally, we found that clinician gender influenced pain scores with male clinicians assigning lower pain scores overall compared to female clinicians. Patients who were assigned higher pain scores were more likely to be assigned a pain management plan and to be prescribed opioids. Other important factors that emerged to explain differences in pain management plans assigned included the patient's age and body condition. Even accounting for these covariates, the effect of breed and transfer service remained robust. The effect of transfer service intuitively made sense as the patients assigned higher pain scores and more frequently prescribed pain management were transferred to services who routinely see cases that may be considered more painful (e.g., orthopedic and soft tissue surgery, ophthalmology). Follow up analyses from an independent evaluation of presenting conditions revealed that breed effects do not appear to be driven merely by disproportionate distribution of painful presenting conditions, suggesting that differences in the assessment and treatment of pain across patients of different breeds do indeed exist. Some of the present findings aligned with previously reported pain sensitivity ratings provided by veterinarians for certain breeds (32). However, our hypothesis was only partially supported as this was not the case for other breeds, suggesting that veterinarians’ pain sensitivity beliefs alone do not explain the breed differences in pain recognition and treatment found here.

**Figure 6:**
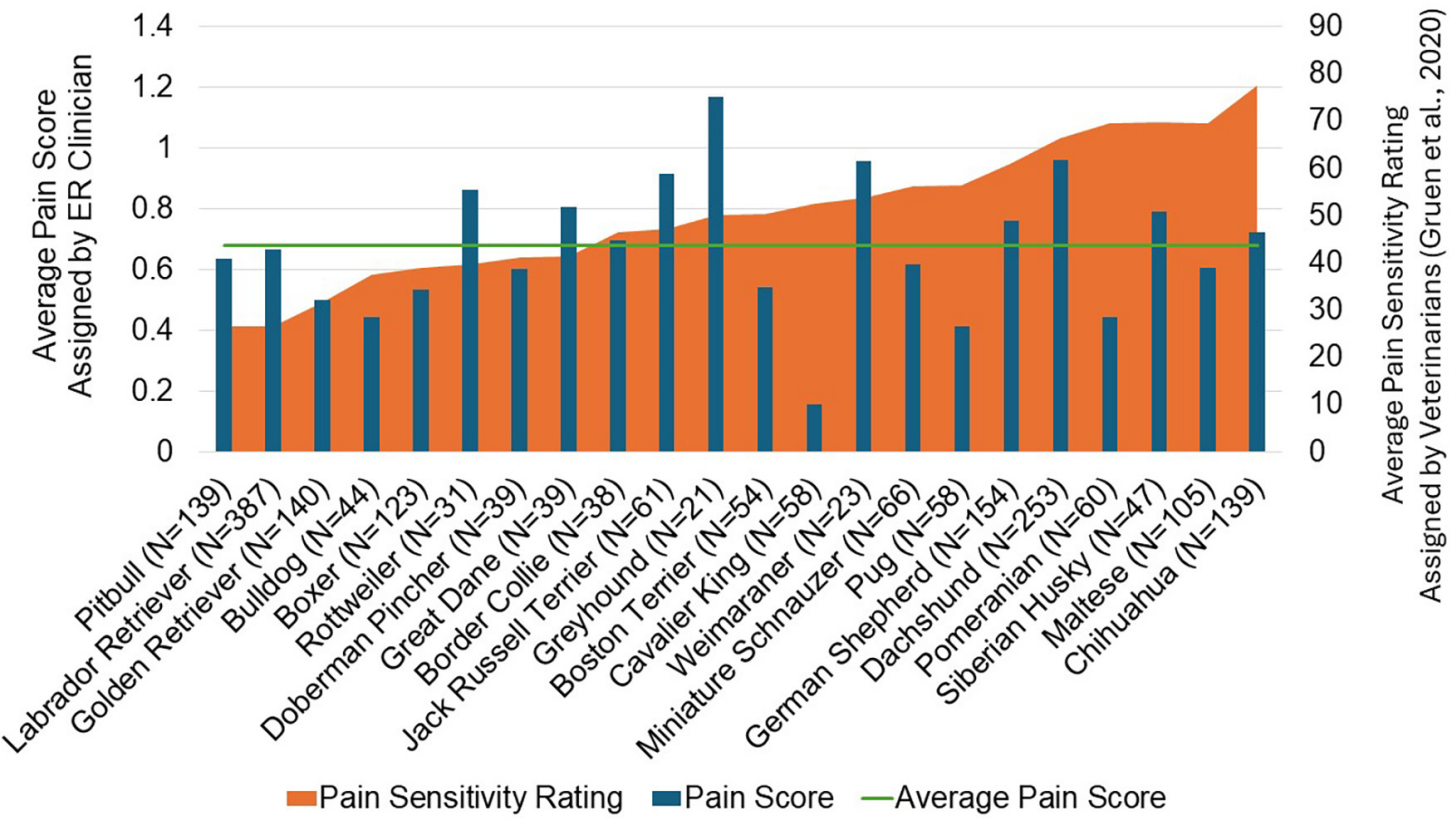
A comparison of the average pain score assigned by emergency room clinicians in the present study and the average pain sensitivity assigned by veterinarians in the gruen et al. (32) study for 22 breeds. The mean values are displayed for each breed. The horizontal line represents the average pain score across all breeds for the population of patients in the present study.

**Figure 7:**
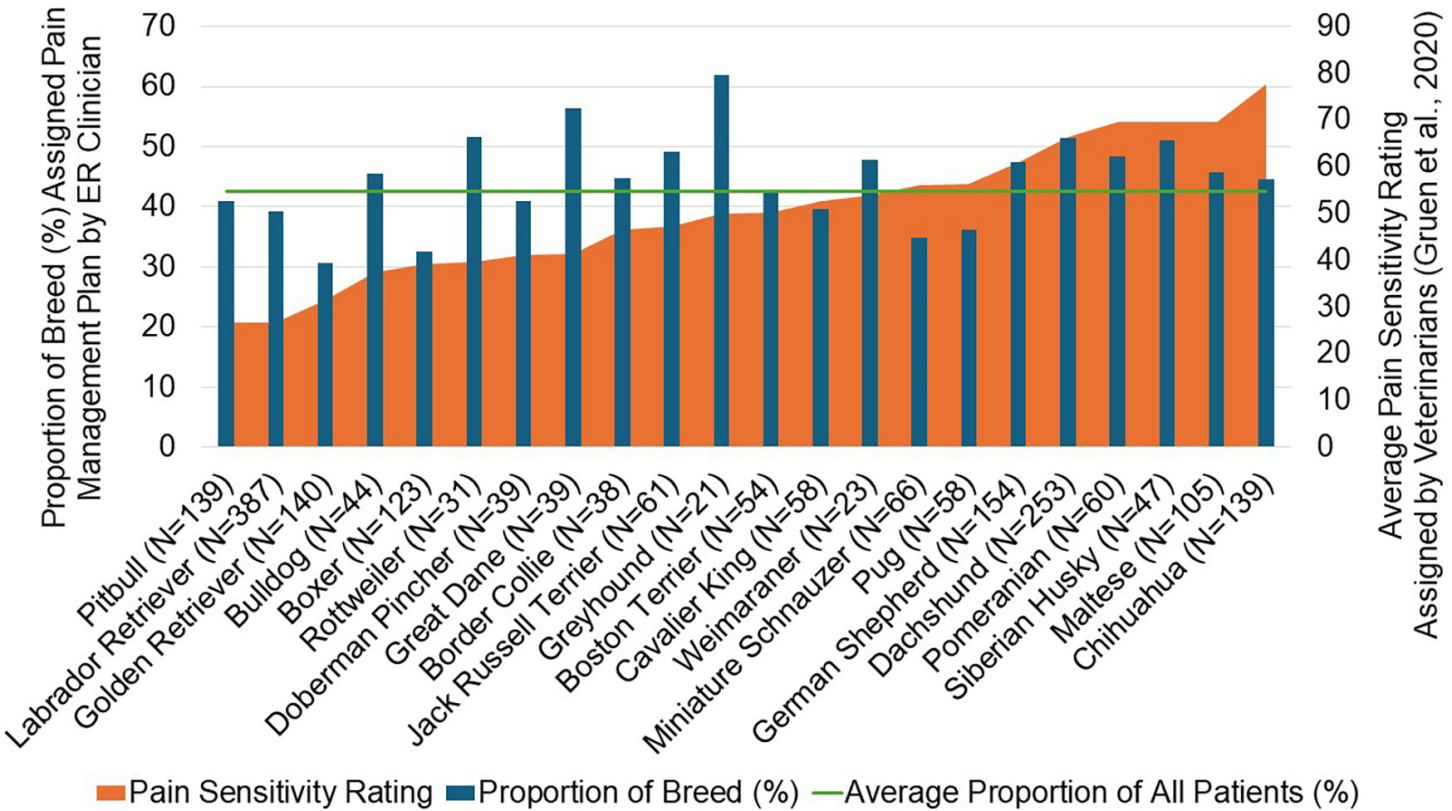
A comparison of the proportion of patients assigned a pain management plan by emergency room clinicians in the present study and the average pain sensitivity rating assigned by veterinarians from the gruen et al. (32) study for 22 breeds. The horizontal line represents the average proportion of patients assigned pain management plans across all breeds for the population of patients in the present study.

From the 28 original breeds evaluated in the Gruen et al. study (32), 22 breeds were included in the final data set due to limited numbers of patients for certain breeds. This provided a basis for comparisons of pain scores and pain management plans assigned in the present study, and pain sensitivity ratings previously reported by veterinarians for these breeds (Figures 6, 7). We found that some breeds’ pain scores aligned well with the breed-specific beliefs held by veterinarians. For example, Golden Retrievers, Boxers, and Doberman Pinchers were all rated as having low pain sensitivity by veterinarians and indeed, these breeds had lower than average pain scores and a lower proportion of these breeds received pain management plans. On the other end of the spectrum, Chihuahuas, German Shepherds, Siberian Huskies, and Dachshunds were rated as highly sensitive to pain by veterinarians and were assigned higher than average pain scores and a higher proportion of these breeds received pain management plans. Interestingly, veterinarians have demonstrated strong beliefs pertaining to Pitbulls’ and Labrador Retrievers’ pain sensitivity or lack thereof, as they consistently rate these breeds as the least sensitive to pain. In the present study, Pitbulls and Labrador Retrievers did have lower than average pain scores and lower proportions of these breeds received pain management plans. However, the pain scores and pain management plans assigned were higher than anticipated if using only pain sensitivity ratings as a guide, supporting individual characteristics influencing pain scoring.

It remains possible that the breed differences observed in pain scoring and pain management plans stem from certain breeds presenting more frequently for painful conditions or from ER clinicians relying on known breed-disease associations when assessing pain. To explore these possibilities, we conducted an independent secondary evaluation in which three clinicians, blinded to breed, rated presenting complaints as painful or not painful. Our findings revealed inconsistencies among clinicians in classifying conditions as painful, indicating that pain scores and pain management plans were not solely determined by the presenting condition. However, it is unsurprising that breeds like Dachshunds received higher-than-average pain scores and were more frequently assigned pain management plans, as their presenting complaints were often rated as painful. Indeed, conditions such as intervertebral disc disease (IVDD), which is more common in Dachshunds, were consistently classified as painful by the raters. In contrast, two of the three clinician raters classified the majority of conditions presented by Golden Retrievers as painful, yet ER clinicians assigned this breed lower-than-average pain scores and were less likely to provide pain management. This discrepancy further supports the notion that breed effects on pain scoring and pain management cannot be fully explained by presenting complaints or breed-disease associations alone.

Another possibility is that clinicians may rely more on breed-specific beliefs when there is uncertainty regarding the patient's pain. Our group has shown that students more frequently used breed-specific language to identify pain when unsure about a patient's condition (33). In the present study, the disagreements in pain ratings, by three clinicians blinded to breed, for presenting conditions further demonstrates how complicated determining pain can be; this finding is fascinating and worthy of additional research. Disagreements were particularly evident for conditions like vomiting or coughing, which may depend on clinician experience with these symptoms in their own life, their observation of patients experiencing these symptoms, or other undetermined factors. In cases more clearly associated with pain, such as fracture or trauma, raters agreed with one another. This is also found in studies of human medicine, where physicians show higher agreement for clear (vs. ambiguous) cases (10, 43, 44). In addition to presenting conditions, veterinary clinicians are necessarily relying on behavioral characteristics of dogs when assessing pain and the need for pain management. We have shown that veterinarians strongly endorse the idea that temperament influenced their ratings (32), and varying views on dog anxiety, fear of pain, and vocalization (45) may have played a role in the pain scoring and pain management plan differences found here.

Several of our findings align with clinical experience and provide support for the veracity of our findings. As expected, dogs with higher pain scores were more likely to receive a pain management plan, and were more likely to receive opioid pain management, regardless of breed. Two unexpected findings were the overall low pain scores assigned to dogs presenting to the ER (even those being transferred to services where painful conditions are common), and the lack of NSAIDs prescribed to dogs presenting with potentially painful conditions. Under-recognition of pain has been reported previously (46) but remains an area where further research is needed. Pain scoring systems may lack sensitivity or may not be used as recommended in all settings. In addition, transfer sheets may be written right after presentation, or toward the end of the shift, meaning that clinicians are relying on their memory of the patient if a pain score was not recorded immediately. This temporal disconnect between seeing the patient and writing their summary may influence pain scoring, though this has not been shown in veterinary medicine. The lack of NSAID use was surprising but potentially explained by the recommended use of opioids when patient status or diagnosis is unclear, as is common in the ER (47), the generally low potential for opioid-related side effects, and the desire to not administer a treatment that would hinder work-up from the receiving service, or risk an adverse event in a patient where the physiological status may not be fully known. Further, this study included only those animals who were hospitalized for transfer to another service; patients seen on emergency and discharged may have received NSAIDs and are not represented here. While some studies have also shown that opioids were the most prescribed analgesics in the veterinary hospital (48, 49), others have found greater use of NSAIDs than we found here (50, 51). Whether these reflect regional differences or reflect the presenting populations and conditions is unknown.

Additional factors that emerged include clinician gender, the patient's age group, and the patient's body condition score. In general, male clinicians provided pain scores that were lower than those provided by female clinicians. This aligns with previous findings showing that male veterinarians are less likely than their female colleagues to recognize pain in their patients (39–42). Additional studies of analgesic use in veterinary hospitals have found that female clinicians were more likely to provide analgesics than male clinicians (39, 41, 50, 52), and this is mirrored in human medicine, where it has been shown that female physicians are more likely to prescribe analgesics than male physicians (53). However, in our study we did not find that the difference in pain scores extended to differences in provision of pain management plans. While there are many potential reasons for gender differences in pain management that have been discussed in the human literature, there is the additional potential for clinician gender to influence the display of pain signs in animals. In rodents, several well-controlled studies have demonstrated that mice and rats will inhibit signs of pain when in the presence of male experimenters, with even just the presence of a t-shirt worn by a male sufficient to evoke this difference (54). Changes in patient demonstration of pain when evaluated by clinicians of different genders has never been shown in companion animal practice, but future work may wish to evaluate pain scoring in a more standardized setting. Regarding life stage, it is possible that senior dogs may have other concurrent painful conditions or a more complicated medical history that contributed to the provision of a pain management plan, despite not having higher pain scores than adult dogs. The study by Hansen and Hardie (48) also found that dogs over 8 years of age were more likely to be given an analgesic than adult or juvenile dogs. The factors impacting body condition score's effect on pain management plans are unclear. Holding all else constant, the odds of receiving a pain management increases by 9.5% for each unit increase in BCS. It is possible that clinicians may be worried about underconditioned dogs (BCS 1–3) not being as robust and able to handle analgesia, though this would not explain why overconditioned dogs were more likely to receive a pain management plan than dogs with ideal BCS. It is possible that having a higher BCS may influence the animal's behavior (e.g., increased respiratory challenges, difficulty moving) in a way that might make clinicians more concerned about pain status, but this is only conjecture. Future work will be needed to more fully explore this finding and the distribution of presentations among dogs with varying BCS.

This is the first study to specifically evaluate the effect of breed on pain scoring and management in this way. Size has been included in previous studies of pain scoring and analgesia use in hospitals (48) but breed has not been directly studied. This study also used a real-world setting, large sample size, and a comprehensive evaluation of all types of ER visits rather than a single presenting complaint. However, in the future it may be beneficial to look across one relatively objectively painful condition to minimize the number of confounding factors that can affect pain scoring. This study was limited to a single university ER, potentially limiting the generalizability across practices more broadly. While the university ER may see a high proportion of complex cases requiring advanced diagnostics or specialist care, it also serves as a first-opinion ER visited by the surrounding community, and represents a wide range of presentations, likely reflecting the scope and variety of cases seen in other emergency practices. Regional differences in breed popularity or presentation to the ER may also limit generalizability. The local pain management and prescribing culture may have influenced practice, but this setting also allowed for evaluation across many different clinicians, which may not have been possible in another setting. As this is a retrospective evaluation of records spanning several years, we cannot verify the training of individuals in the use of the pain scale, though this scale had been in practice at this ER for many years. Still, misinterpretation of the scale could influence findings. While these limitations should be kept in mind in interpreting the results, the size of data set and robustness of the findings mean additional research is warranted. In particular, understanding what influences whether a clinician regards a given presentation as painful, what behavioral signs they use when evaluating patients, and whether those behavioral signs are influenced by dog breed would provide valuable insight.

In conclusion, this study demonstrated that veterinarians in an ER setting assigned pain scores that were influenced by breed and receiving service. Certain breeds were assigned pain scores lower than average, while other breeds were assigned higher than average pain scores despite a lack of evidence that these breeds presented with less or more painful conditions overall. Evidence from previous work suggests that holding beliefs about pain sensitivity in dogs of different breeds is not useful in a clinical setting as experimental paradigms show that pain sensitivity ratings do not fully explain differences in pain response thresholds (55). Future work will further evaluate the behaviors clinicians rely on as an indication of pain in their patients, what conditions veterinarians believe to be associated with pain, and whether breed affects pain scores when restricted to a standardized set of presenting complaints. Future work is also needed to explore the differences in pain scoring from male and female clinicians, and how much of this is influenced by differential patient behavior. While, overall, pain scoring was not fully aligned with pain sensitivity ratings, and higher pain scores were associated with provision of a pain management plan regardless of breed, clinician awareness of the potential for breed to influence pain scoring may be useful in a clinical setting as we continue to refine medical decision-making processes and pain education.

## Data Availability

The raw data supporting the conclusions of this article will be made available by the authors, without undue reservation.
