# Supplementary material for: Tailoring treatment: dog breed status influences pain assessment and treatment in emergency veterinary care
Source: Front Pain Res (Lausanne). 2025 Nov 17;6:1589082. doi: 10.3389/fpain.2025.1589082 (PMC12665740; doi:10.3389/fpain.2025.1589082)
Supplement: Supplementary file 1 [file Datasheet1.docx]

Supplementary Material

# Supplementary Material

The pain scoring system was formally introduced to faculty, residents and interns through didactic sessions as part of an introductory seminar series. New residents and interns continue to receive this formal training on the pain scoring system. Since its implementation, supervising senior clinicians have routinely reviewed trainee assessments, and ongoing instruction by senior ER faculty has ensured consistent training for new hires.

# Supplementary Tables and Figures

## Supplementary Tables

**Supplementary Table 1. Patient breed labels used in medical records that were condensed into a single breed label for inclusion in the final data set.**

| **Breed Label in Final Dataset** | **Patient Breed Labels Used in Medical Records** |
| --- | --- |
| Pitbull | Pitbull, American Bulldog, American Staffordshire Terrier, Staffordshire Bull Terrier |
| Coonhound | Coonhound, Blue Tick Coon Hound |
| Belgian Malinois | Belgian Malinois, Malinois |
| Springer Spaniel | American Springer Spaniel, English Springer Spaniel |
| Spinone Italiano Dog | Spinone Italiano Dog, Italian Spinone |
| Settter | English Setter, Irish Setter |
| Pointer | Pointer, English Pointer |
| Bulldog | Bulldog, English Bulldog |

## Supplementary Figures

**
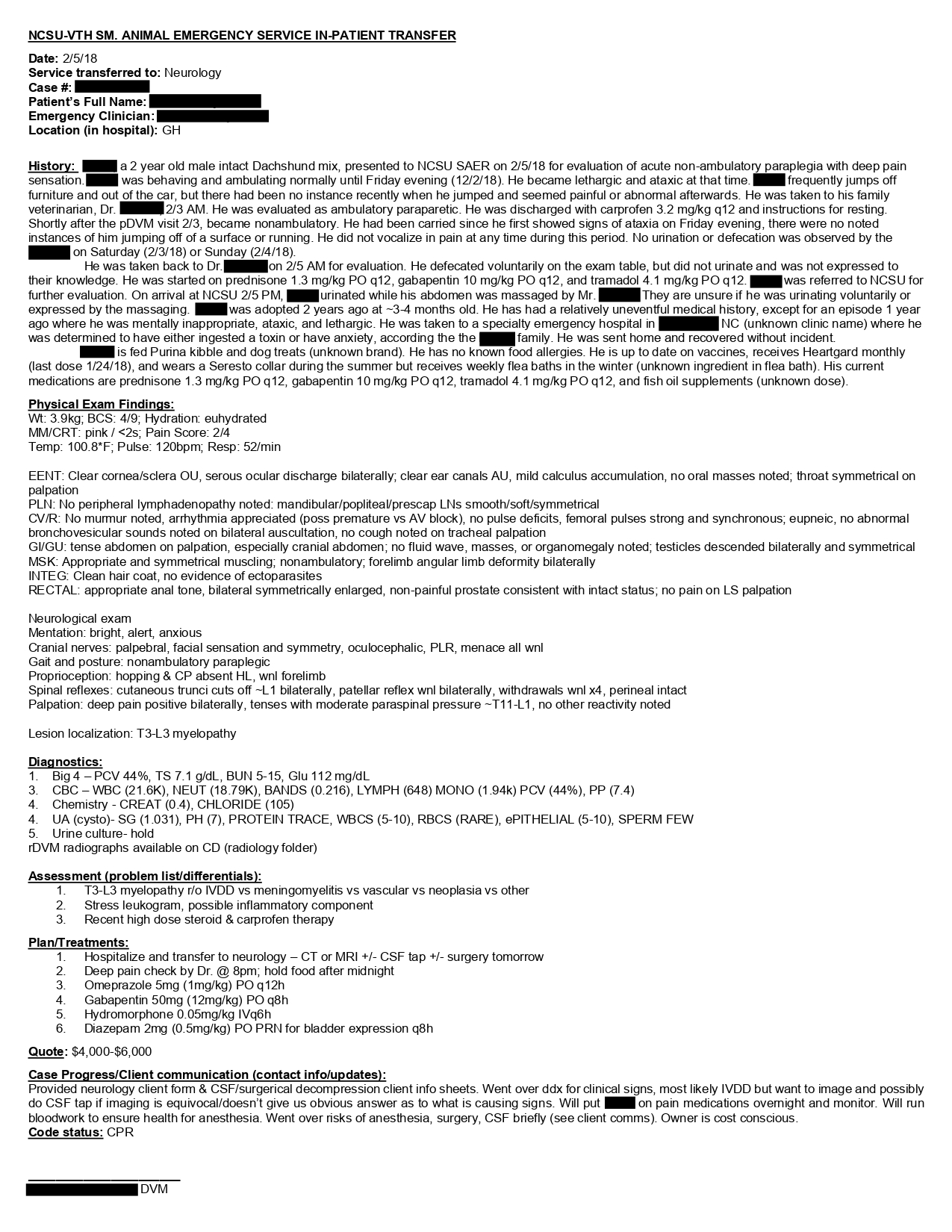
**

**Supplementary Figure 1.** **An example ER transfer sheet created for a patient in the NCSU ER.** This document summarizes the patient’s clinical data for the receiving hospital service that the patient is transferred to. Re-identifying information was redacted for privacy purposes.


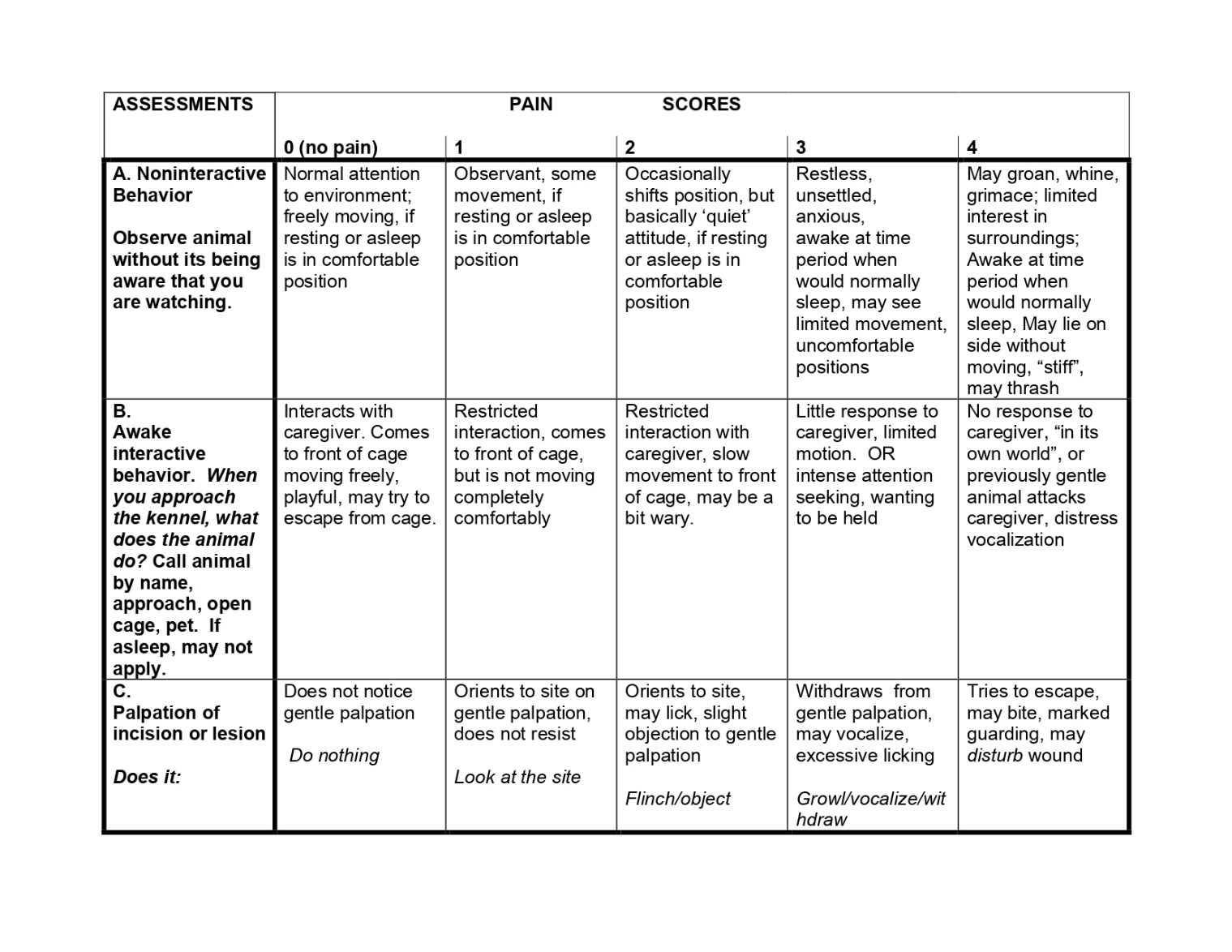


**Supplementary Figure 2**. **Pain scale for hospitalized patient.** Clinicians performed the observations in this order. Clinicians should have noted on record if gentle palpation is contraindicated. Clinicians recorded the highest score.


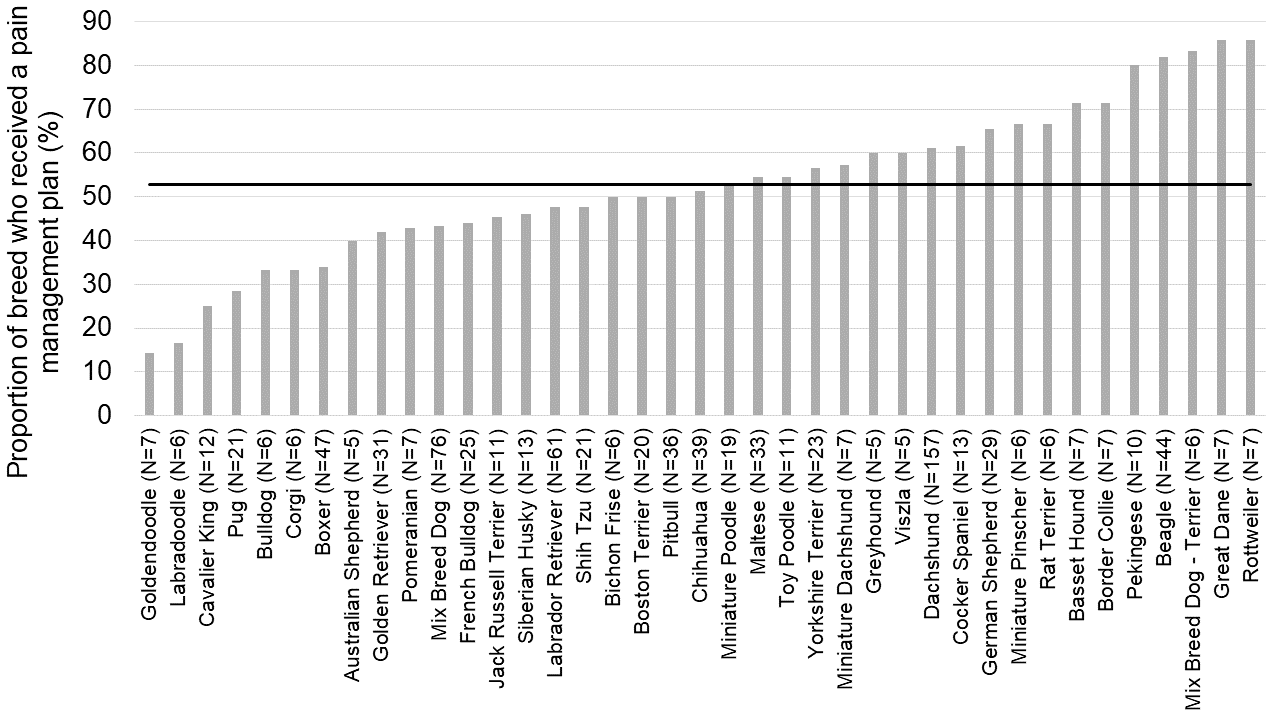


**Supplementary Figure 3.** **Proportion of patients assigned a pain management plan by emergency room clinicians for each breed that were transferred to neurology from the emergency room.** The horizontal line represents the average proportion of patients assigned pain management plans for this population of patients.


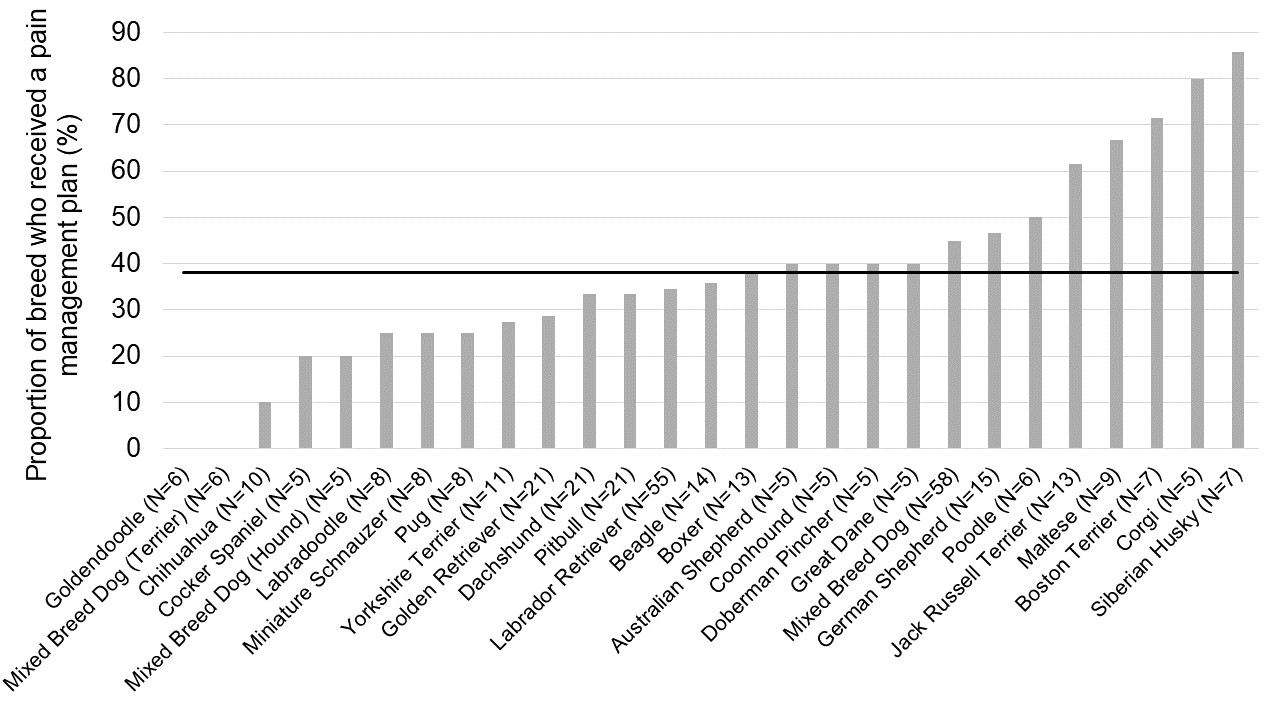


**Supplementary Figure 4. Proportion of patients assigned a pain management plan by emergency room clinicians for each breed that were transferred to triage from the emergency room.** The horizontal line represents the average proportion of patients assigned pain management plans for this population of patients.
